# The Typical Triad of Idiopathic Normal Pressure Hydrocephalus in a 62-Year-Old Male

**DOI:** 10.7759/cureus.18090

**Published:** 2021-09-19

**Authors:** Naela B Alamoudi, Ruba K Alnajim, Dunya AlFaraj

**Affiliations:** 1 Emergency Medicine, College of Medicine, Imam Abdulrahman Bin Faisal University, Dammam, SAU; 2 Emergency Medicine, Imam Abdulrahman Bin Faisal University, Dammam, SAU

**Keywords:** normal pressure hydrocephalus, lumbar puncture, triad, idiopathic, case report

## Abstract

Normal pressure hydrocephalus (NPH) is a rare pathological condition of the brain in which the ventricles are enlarged due to cerebrospinal fluid accumulation and is associated with normal opening pressure on lumbar puncture with a large-volume cerebrospinal fluid (CSF) tap. This results in three classical symptoms: mental impairment, gait disturbance, and urinary or fecal incontinence. We present a case of idiopathic NPH in which a 64-year-old retired man with diabetes was brought to the emergency department after recurrent previous falls. The patient complained of an unsteady gait and presented with the typical triad of NPH which is mental impairment, gait disturbance, and incontinence. The patient was a known diabetic, and his gait was characterized by shuffling, bradykinesia, and mild drifting toward the right side. A head computed tomography scan revealed brain tissue volume loss, with disproportionate dilation of the lateral and third ventricles. A lumbar puncture with a large-volume CSF tapping produced normal opening pressure (18 mmHg); thus, the diagnosis of NPH was made. The patient underwent shunt surgery, and his balance and memory improved significantly after the procedure. Also, no event of fecal incontinence occurred. NPH resembles several neurodegenerative disorders. Due to this, it can be difficult to diagnose. Emergency physicians, as frontline healthcare providers, may encounter such cases.NPH should be considered in patients presenting with an unsteady gait, memory impairment, and urinary or fecal incontinence by taking a detailed history and conducting a physical examination to prevent future complications.

## Introduction

Hydrocephalus is a condition characterized by the buildup of excess cerebrospinal fluid (CSF) due to either an obstruction within the CSF flow pathway, excess fluid production, or reabsorption defect in the arachnoid villi, leading to its accumulation in and enlargement of the cerebral ventricles [1]. Idiopathic normal pressure hydrocephalus (iNPH) is considered one of the common forms of communicating hydrocephalus in adults [2]. In a population-based study, it was found that the prevalence of iNPH of many elderly people who have clinical and imaging signs of iNPH and need to be evaluated further increased over the past years from 0.2% to 6.0% within the age group 70 to 80 years and is equal for both genders. According to the findings of this report, the number of people with untreated iNPH patients is much higher than the number of treated patients. Given the negative effects of untreated cases, it is critical to raise awareness and develop the diagnostic and treatment options for such diagnoses. Furthermore, if the general population's life expectancy was increased, it is possible that the number of people with iNPH will rise as well [2].

NPH is characterized by a combination of the classical clinical triad of gait disturbance, urinary or fecal incontinence, and memory impairment. In general, several studies have shown that the presence of the cardinal symptoms, in addition to other features, is essential for NPH diagnosis. However, cognitive impairment remains indistinct and even concealed [3]. Furthermore, NPH is typically characterized by an increase in the size of the cerebral ventricles and normal pressure once the spinal tap is applied [3].

Many elderly people suffer from symptoms that constitute the triad of iNPH and resemble several neurodegenerative disorders. The commonest differential diagnoses are Parkinson’s disease, Alzheimer’s disease, and vascular dementia. Thus, iNPH can be difficult to approach. The following findings make the diagnosis of iNPH less likely: Intracranial pressure greater than 25 cmH2O (this rules out iNPH by definition), under the age of 40 (iNPH unlikely), signs and symptoms that are asymmetrical or fleeting, cortical deficits such as aphasia, apraxia, or paresis, progressive dementia without gait disturbance (regardless of ventricles enlargement), and lack of symptoms development (a point of concern, as many authors differ on how long signs should be shown in advance). It is one of the neurological conditions that is challenging for some physicians to accurately diagnose and since clinical and radiological results alone are often insufficient to differentiate iNPH from other causes of subcortical dementia, investigations are often needed. Due to this, iNPH can be difficult to diagnose. 

The superior treatment option is by surgically applying a ventriculoperitoneal (VP) shunt [4], It was recommended to “test CSF hydrodynamics to demonstrate either that the patient has the potential to respond to shunt surgery or that the patient has abnormal CSF hydrodynamics that is consistent with hydrocephalus” [2]. Lumbar puncture or lumbar drain trial are mostly performed prior to surgery. In general, if the patient clinically improved following these procedures, which is assessed through several parameters including measures of gait speed, stride length, and the number of steps it takes to turn 180 or 360 degrees and evaluated either by clinicians through gait assessment 30 to 60 minutes after the procedure or by patients themselves and family members, there is a better outcome after the shunt surgery [5]. A study revealed that 60%-80% of patients with iNPH who were treated with a VP shunt improved after the surgery. However, regular follow-ups are required to detect any changes and avoid shunt infections [2].

The current case report illustrates the case of a patient who presented to the emergency department (ED) with the typical triad of iNPH and highlights how the patient was assessed to reach a definitive diagnosis.

An abstract of this case report has been presented in the 1st Bahrain Emergency Medicine Conference on October 10, 2019, and the 5th Saudi Society of Emergency Medicine’s International Conference on February 9-11, 2020, and published in the Saudi Journal of Emergency Medicine on February 8, 2020 [6].

## Case presentation

A 64-year-old man (a retired chemical engineer) with diabetes was brought to the ED by his wife after a fall the day before. The patient presented to the hospital with bruises on the head and chest due to the fall. Although the patient had recurrent visits to the ED because of recurrent previous falls, investigations were not done before the date of this most recent presentation; he was only reassured and discharged. On the current visit, the triage doctor sent the patient to a monitored bed because of head trauma and old age. When the wife was asked about the fall history, she revealed that the patient had recurrent falls in the past three months because of an unsteady gait. Apart from this, there was no syncope, nausea, vomiting, body weakness, seizure, headache, dysarthria, or altered level of consciousness. The patient had a diabetic foot and a three-month history of proliferative diabetic retinopathy in both eyes. The patient and his wife thought his walking problem was a complication of his diabetic foot. Hence, he was receiving physical therapy for that, but his condition kept worsening with time. The patient’s wife reported three events of fecal incontinence and denied urinary incontinence. She also reported forgetfulness over the last two months: “He always forgets his mobile and keys and keeps asking where we are going to, saying, ‘I just want to make sure of that.’” The rest of the medical history was noncontributory.

The patient was vitally stable (heart rate = 99 beats per minute (bpm); blood pressure = 133/77 mmHg; respiratory rate = 19 bpm; O_2_saturation = 99% in room air). On physical examination, the patient’s gait was shuffling and bradykinetic, with mild drifting toward the right side. His pupils were equally reactive to light, and all the cranial nerves were normal. Full sensation and strength were evident, and all reflexes were intact.

**Figure 1:**
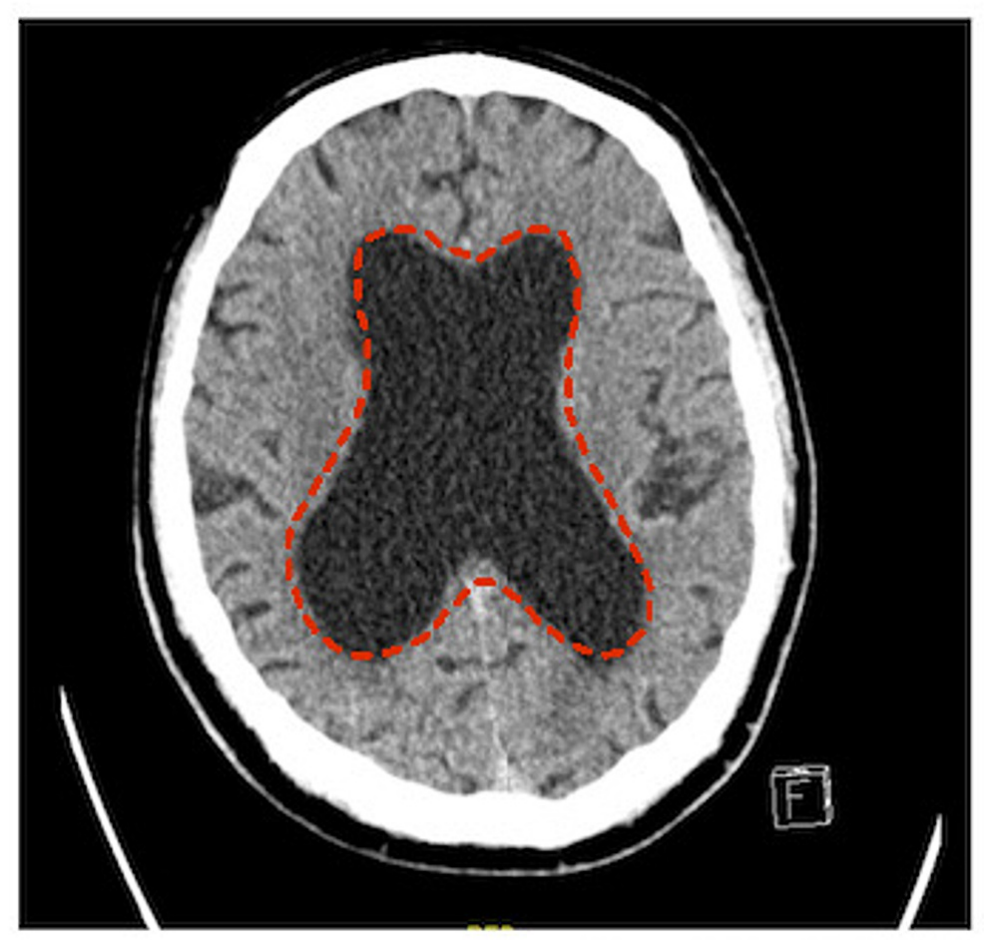
Cross-sectional computed tomography scan of the brain showing dilatation of the lateral ventricles (dashed border)

Blood work was performed and found to be unremarkable (complete blood count, with differentials, as well as liver function, renal function, electrolytes, venous blood gas, and random blood sugar tests). A non-enhanced axial computed tomography scan of the brain was performed and revealed brain tissue volume loss with disproportionate dilation of the lateral and third ventricles, with no obvious obstructive lesions (Figure 1). Subtle periventricular hypodensity was also seen. There was no evidence of acute territorial infarction or acute hemorrhage. No mass effect or midline shift was observed. The posterior fossa structures were unremarkable. The visualized orbital structures and paranasal sinuses were within the normal limits, but a redundant optic nerve sheath complex was seen bilaterally. 

The case was referred to neurosurgery in which they performed a lumbar puncture with 40 ml of CSF tapping and produced normal opening pressure (18 mmHg) (normal range: 5-18 mmHg or 70-245 cmH2O) [4]; thus, the diagnosis of NPH was made. The patient underwent shunt surgery. Seven months later, he visited the hospital for follow-up. He was evaluated, and his gait and memory were found to have significantly improved after the procedure. Also, no fecal incontinence events occurred. 

## Discussion

NPH is a rare neurological disorder compared to other causes of dementia in older adults with a variable incidence in different studies ranging from 2 to 20 per million per year [5].

It has been linked to a number of possible mechanisms. The iNPH patients have a higher prevalence of vascular risk factors when compared to age-matched controls with and without dementia. Furthermore, because three-quarters of NPH patients also have vascular dementia or Alzheimer's disease, distinguishing NPH from other diseases can be challenging. Many elderlies suffer from both motor and cognitive problems, and sometimes urinary incontinence.

Intracranial pressure more than 25 cmH2O, cortical symptoms (aphasia, apraxia, or paresis), Alzheimer's disease that is not accompanied by a gait disorder (also with enlarged ventricles), and age under 40 years are all symptoms that might lead to a diagnosis of NPH. Moreover, peripheral neuropathy, spinal stenosis, inner ear illness, persistent alcoholism, and vitamin B6 and B12 deficiency are all considered to be differential diagnoses of gait problems. Several forms of dementia are included in the differential diagnosis of cognitive impairments. Clinical and radiological findings are the sole way to distinguish NPH from other forms of subcortical dementia; therefore, more intrusive testing is required [7].

From this association, chronic periventricular ischemia, intraventricular pulse pressure, and systolic hypertension cause ventricular enlargement as a result of normal intracranial pressure fluctuations. Chronic periventricular ischemia also increases ventricular wall compliance. Venous system resistance may be increased by periventricular ischemia; thus, CSF absorption will be reduced, resulting in ventricular enlargement. Another proposed mechanism is the increased pressure of the central venous system by the retrograde flow in the internal jugular veins during a Valsalva maneuver. It was also suggested that there is a decreased CSF absorption, and, for that reason, shunt surgery is done for these patients. Ventricular enlargement may also be caused by neurodegeneration, such as Alzheimer's disease [5].

This is a case of a patient with the triad of iNPH-gait disturbance, cognitive impairment, and incontinence. A detailed history revealed the symptoms’ chronology, which led us to consider such a diagnosis. The gradual and progressive onset of gait disturbance was the first and the most prominent manifestation, followed by early memory impairment and fecal incontinence. Since the opening pressure at the lumbar cistern was normal in this patient, there was a notable negative impact in certain signs and symptoms related to the increased intracranial pressure, including headaches, nausea and vomiting, papilledema, and visual loss. The risk factors in the current presented case included diabetes mellitus and the age of above 60 years.

Several differential diagnoses can be attributed to the typical three symptoms of NPH; hence, misdiagnosis is possible. One of the diagnoses is Parkinson's disease dementia since the patient had bradykinesia. However, additional parkinsonian signs such as tremor and rigidity need to be present, and cognitive impairment and dementia present in the late stages of the disease. A key feature that differentiates dementia with Lewy bodies from NPH is the presence of psychotic features, cognitive fluctuations, and rapid eye movement behavior, even though the gait abnormality in this disease may resemble NPH. In addition, progressive supranuclear palsy (PSP) is a common reason for referral for possible NPH. Even though the voluntary gaze impairment is a hallmark, it appears late after years. Speech and swallowing difficulties are clinical findings for PSP. Another possible differential diagnosis is multiple system atrophy (MSA), in which they will have bladder or bowel dysfunction and other autonomic dysfunction features and cerebellar ataxia. The presence of additional autonomic features such as impotence in men, abnormal sweating and postural intolerance, laryngeal stridor, dysarthria, and cervical dystonia helps in guiding the diagnosis toward MSA rather than NPH [5]. Furthermore, other diagnoses, such as common dementia disorders, corticobasal degeneration, and neurosyphilis, can be considered [4,8]. However, conditions such as orthopedic problems, frequent hypoglycemic attacks [8], brain/spinal problems [4], subarachnoid hemorrhage, meningitis, brain surgery, radiation or traumatic brain injury, stroke, and spinal stenosis should be excluded, as they cause secondary NPH [2,3].

Neuroimaging, which is magnetic resonance imaging (MRI) or computed tomography (CT) scan, is needed to demonstrate enlarged ventricles and rule out ventricular obstruction and other causes before making the diagnosis [4]. Although both tests will show the ventricular and sulcal size, MRI is superior to CT because it can exclude other etiologies. CT scan is the initial test ordered in all patients and can exclude NPH, and it is considered an appropriate screening tool in patients who cannot have an MRI [5]. Furthermore, lumbar puncture should also be considered to document normal or mild elevation of CSF opening pressure (5-18 mmHg or 70-245 cmH2O). Because shunt surgery might be appropriate, the probability that a patient would benefit from it should be evaluated using a large-volume CSF tap (30 to 50 ml of CSF). If gait symptoms were improved by one to three hours after the procedure, which is evaluated by recorded timed walks done, there is a high likelihood that the patient will respond to a CSF diversion procedure [4]. Gait speed, stride length, and the number of steps required to turn 180 to 360 degrees are all common parameters in assessing the gait before and after the procedure [5]. Nevertheless, if the results after the CSF tap are equivocal or there is no response to large-volume CSF tap while there is still a high index of suspicion, then external lumbar drainage, infusion testing, and assessment of CSF dynamics should be considered [4].

Patients with iNPH have a good prognosis following CSF shunt surgery. Toma et al. reported improvement of gait symptoms in ≥ 85% of patients, cognitive symptoms in up to 80% of patients if performed early, and incontinence in up to 80% of patients [9]. In addition, as iNPH is treated with a shunt system, it can cause surgical complications and other issues that could develop days or years after treatment. The most serious complication is subdural hematoma following shunt insertion, in which patients present with deterioration and confusion. Other complications might include VP shunt infection caused by *Staphylococcus* and long-term complications such as vascular disease and cognitive impairment (e.g., dementia). As a result, the complication rate has been reported as 38% [9]. There is a possibility for further progressive deterioration if shunt surgery is not performed in such patients [10].

Conn noted in his report that patients themselves and their families, not physicians, have the final decision of whether or not shunt surgery should be undertaken [10].

## Conclusions

A diagnosis of NPH could be missed by many clinicians since the symptoms resemble other neurodegenerative disorders. Emergency physicians may encounter such undiagnosed cases. Obtaining a proper history and the evaluation of the presence and timing of triad symptoms appearance plus the assessment of other neurological features help in distinguishing NPH from other differential diagnoses. The diagnosis of NPH should be considered while taking a detailed history to prevent future complications. CT scan is the initial test of choice and is a proper screening test for exclusion. These patients will have a good prognosis and major improvement in their clinical symptoms following shunt surgery.

## References

[1] (2020). Hydrocephalus. https://www.aans.org/Patients/Neurosurgical-Conditions-and-Treatments/Hydrocephalus.

[2] Williams MA, Malm J (2016). Diagnosis and treatment of idiopathic normal pressure hydrocephalus. Continuum (Minneap Minn).

[3] Shprecher D, Schwalb J, Kurlan R (2008). Normal pressure hydrocephalus: diagnosis and treatment. Curr Neurol Neurosci Rep.

[4] Grünewald R (2020). Normal pressure hydrocephalus. https://bestpractice.bmj.com/topics/en-gb/712.

[5] (2020). Normal pressure hydrocephalus. https://www.uptodate.com/contents/normal-pressure-hydrocephalus.

[6] Alamoudi NB, Alnajim RK, Alfaraj D (2020). The typical triad of idiopathic normal pressure hydrocephalus in a 62-year-old male: a case report. SJEMed.

[7] Oliveira LM, Nitrini R, Román GC (2019). Normal-pressure hydrocephalus: a critical review. Dement Neuropsychol.

[8] Iino K, Yoshinari M, Yoshizumi H, Ichikawa K, Iwase M, Fujishima M (2000). Normal pressure hydrocephalus in diabetic patients with recurrent episodes of hypoglycemic coma. Diabetes Res Clin Pract.

[9] Toma AK, Stapleton S, Papadopoulos MC, Kitchen ND, Watkins LD (2011). Natural history of idiopathic normal-pressure hydrocephalus. Neurosurg Rev.

[10] Conn HO (2007). Normal pressure hydrocephalus: a case report by a physician who is the patient. Clin Med (Lond).

